# Dignity at stake – relatives’ experiences of influencing dignified care in nursing homes

**DOI:** 10.1186/s12913-023-09175-3

**Published:** 2023-02-23

**Authors:** Trude Anita Hartviksen, Jessica Aspfors, Lisbeth Uhrenfeldt

**Affiliations:** 1 Center for Care Sciences, North, UiT, The Arctic University of Norway, Tromsø, Norway; 2 Faculty of Education and Arts, Nord University, Bodø, Norway; 3 Faculty of Nursing and Health Sciences, Nord University, Bodø, Norway

**Keywords:** Dignity, Nursing home, Relatives, Focus groups, Secondary analysis, Knowledge-based practice, Quality improvement

## Abstract

**Background:**

Dignity, in the care of older nursing home residents, has been an increasingly part of the public discourse the recent years. Despite a growing body of knowledge about dignity and indignity in nursing homes, we have less knowledge of how relatives experience their role in this context. This study is a follow-up to a previous study in nursing homes, which gave rise to concern about the relatives’ descriptions of residents’ dignity. The aim of this current study is to critically discuss relatives’ experiences of influencing the dignified care of residents of nursing homes.

**Methods:**

Methodologically, the study is informed by a critical hermeneutic stance, where the analysis is guided by a qualitative interpretive approach and a humanizing framework. This is a secondary analysis that includes data from five semi-structured focus groups from a previous study. The participants were 18 relatives of 16 residents living in two nursing homes in rural northern Norway.

**Results:**

The main theme in this study, preventing missed care when dignity is at stake, is identified when relatives of nursing homes experience that they are able to influence dignified care by (a) pinpointing to prevent missed care and (b) compensating when dignity is threatened.

**Conclusions:**

Despite their stated good intentions to safeguard dignity, relatives of nursing homes experience being alienated in their attempts to change what they describe as undignified and unacceptable practice into dignified care. The relatives’ observations of dignity and indignity are, contrary to what national and international regulations require, not mapped and/or used in any form of systematic quality improvement work. This indicates that knowledge-based practice in nursing homes, including the active application of user and relative knowledge, has untapped potential to contribute to quality improvement towards dignified care.

## Background

Dignity is the first article in the United Nations Universal Declaration of Human Rights [1], and is a key concept in international guidelines for the care of older persons [2]. The concept of dignity is explained and interpreted differently in different sources, where historical references can be traced all the way back to Aristotle [3].

This study draws on Galvin and Todres’ humanizing framework [4], where dignity is explained as: “the affirmation of something valuable in oneself or another as an inheritor of Being” [5], p. 411), and their explication of how such an affirmation can be not only easily ruptured but also restored. This modern existential understanding of dignity recognizes human beings as having inherent dignity [5], which corresponds to how human rights are framed in the ethical guidelines for health personnel, regardless of diversity factors (such as age, color, creed, culture, disability or illness, gender, sexual orientation, nationality, politics, race, or social status) [6]. Galvin and Todres [4] describe the concept of dignified care as an opportunity for health personnel to confirm, by their actions, that another person is valuable while simultaneously being in need of care. Dignified care is further explained as being provided within six existential dimensions (existentials): spatiality, temporality, inter-subjectivity, mood, identity, and embodiment [4].

These six existentials are experienced in a continuum between well-being and suffering. Dignified care is understood as contributing to well-being, whilst missed care could lead to suffering [4]. The concept of missed care describes a situation in which the provision of required healthcare is delayed or omitted [7]. Missed care is described as being triggered by acuity, complexity, the amount of care, and organizational factors, while factors such as appropriately skilled nurses, inadequate staffing, documentation, and communication are less well-evidenced [8]. Although spatiality adds to suffering in the context of feeling imprisoned, exiled, or spaceless, temporality may involve a blocked future, an elusive present, or an absence of respite. Inter-subjectivity can include aversion, alienated isolation, or persecution, while mood describes the potential for suffering through depression, agitation, or restless gloom. Identity incorporates the feeling of being unable, like an object or ‘thing’, or of being fragmented. Embodiment can be experienced as stasis or exhaustion, bodily discomfort or pain, or painfully closing down [4].

From the nursing home (NH) residents’ perspective, dignity is highlighted as representing an ongoing identity process. This process is based on opportunities to be involved in decisions concerning oneself, and is confirmed in interaction with significant others [9]. Although the dignity of residents is recognized as an essential component when health personnel conceptualize the importance of high-quality services in NHs [10], this has been questioned in various contexts [2, 11]. An example of this occurred during the COVID-19 pandemic, when relatives were denied access to NHs. This resulted in concerns regarding the denial of relatives’ opportunities to check the provision of dignified care and to ensure the fulfillment of the residents’ rights to optimal healthcare [12].

### Nursing homes as part of the Scandinavian welfare state model

This study was conducted in Norway, where The Dignity Guarantee is enshrined in the regulations and legislations that apply to municipal healthcare [13, 14]. Similar legislations is found in other Nordic countries, such as Denmark, Finland, and Sweden [15]. As such, dignified care in NHs can be regarded as being embedded in what is known as the Scandinavian (or Nordic) welfare state model, which includes a comprehensive social policy, universal rights, and legislations [16].

Sandvin and Vike [17] argue that care of older persons is the most service-intensive and universal benefit that is established by the welfare state model. Norwegian NHs are primarily publicly financed by taxes and residents’ deductible, and they are generally operated by the municipalities: only 10% are operated by private suppliers [18]. NHs are identified as places where older persons live and receive treatment, training, and help with daily chores, for shorter or longer periods [19]. However, institutionalization is a recognized factor that has a negative influence on older persons’ quality of life [20].

In the 1980s, the welfare state model experienced a political change of course, including several state reforms (exemplified by the NH reform of 1988). The purpose of these changes was to bring public spending under control. Since then, financial incentives have been replaced by frameworks and stronger management through legislations. As in other Western countries, Norway is facing a significant increase in the number of older persons. A number of new assignments have also been imposed on municipal healthcare providers as a result of medical developments and political decisions. This shift is not directly connected to financing. Thus, the state has been criticized for creating expectations among the population, while the municipalities are left to prioritize and distribute limited available resources [21].

### Relatives’ involvement in nursing homes

Relatives of NHs are identified as the resident’s next of kin. These persons could be either family members or significant others [22]. Relatives have been described as acting as the residents’ voice when raising concerns to ensure that their loved ones are treated respectfully and with dignity [23]. Four different roles are framed: (1) hands-on assistance; (2) keeping track of, managing, and negotiating treatment and care; (3) providing socioemotional support; and (4) enhancing the well-being of other residents [24]. Relatives describe experiencing dignity when basic care and spiritual support are provided. This includes the recognition and treatment of symptoms, ensured continuity, respect for the resident’s wishes, and the provision of environmental, emotional, and psychosocial support. It also assumes that the family is kept informed, and that a family understanding is established in the form of a partnership in which relatives are involved and provided with guidance in joint decision-making [25].

There are several examples of how legislations and regulations guide health personnel to involve relatives when the patient is not able to make personal decisions [14, 26, 27]. Relatives’ involvement is central to establishing adapted healthcare services with regard to human dignity and improved healthcare quality [22]. The Norwegian Regulations on Management and Quality Improvement in the Health and Care Service [27] require health personnel to obtain an overview of deviations, with the help of patients’ and relatives’ experiences, and to make use of this knowledge. Nevertheless, relatives that become proxy decision-makers on behalf of residents have described this role as uncertain and distressing [28].

In a systematic review, Pulst et al. [29] show relatives to be an important link in communication, between residents and health personnel, as well as between NHs and hospitals. They found that the degree of involvement of relatives in the decision to hospitalize residents varies from no involvement to full agreement with the relatives’ personal preferences. Conflicts between relatives and health personnel generally occur with regard to the interpretation of the resident’s best interests. Such discussions are experienced by relatives as challenging, emotionally stressful, and uncomfortable [29].

The socio-organizational processes involved when relatives are present in NHs are underlined as a complex area that is in need of more critical investigation [24]. Despite an increasing body of knowledge about dignity and indignity in NHs, there is relatively little knowledge about how this is experienced by residents and relatives [15].

In a previous study, we identified and critically discussed how healthcare middle managers’ (HMMs) development of the capacity and capability for leadership are experienced as influencing quality improvement (QI) in NHs. This was part of a threefold project, which investigated HMMs’ development from macro- [30], meso- [31], and micro- [32] perspectives [33]. The methods in the (third) micro-perspective study included five focus groups comprising 18 relatives of 16 residents living in two NHs [32]. The transcribed data from these five focus groups gave rise to a concern about the relatives’ descriptions of dignified care in NHs. This current study responds to this concern by reanalyzing the data from the same five focus groups and with the same 18 participants, but with a new aim. The aim of this current study is to critically discuss relatives’ experiences of influencing the dignified care of residents of NHs.

## Methods

A critical hermeneutic stance [34] has influenced the design of this qualitative study, in all its research phases. The measures taken to ensure the study’s trustworthiness have previously been disseminated [33]. This study’s starting point reflects the critical hermeneutic process [34, 35]; it has been developed from a concern raised in a previous study about the participants’ unsolicited statements relating to their own experiences of dignified care in NHs. In this secondary analysis, the self-collected data from five focus groups has been re-used in order to investigate a new research question [36]: How do relatives experience influencing the dignified care provided to residents in NHs?

The development and moderation of focus groups is based on an epistemological understanding of knowledge, as justified through several subjectivities and inter-subjectivity, of which the participants’ inter-subjective interaction provides access to their lifeworld, understood as a culturally inherent pre-understanding [34]. This pre-understanding builds on critical reflection that was based on the participants’ and the researchers’ lifeworld’s, during and after the previous study [32]. Truth is thus constructed as a dialogical process between researchers and participants, and is handled by transparency and critical reflection. The critical reflection took place in an inter-subjective dialogue and interaction, where the participants’ lifeworld’s were pre-understood as having been colonized by the system. This is to be seen as a balancing process between the first author (TAH) and the participants, and within the research team, that involves searching for contrasts and accentuating theoretical statements that represent changeable dependent relationships [34].

### Design

The initiation and design of this study were developed with selected parts of the empirical data, and by the same research team, as in the previous study [32]. The research team comprises a collaboration between three Scandinavian researchers (authors) with different professional backgrounds and academic experience.

The participants were initially included in three focus groups. During the analysis process, all participants were invited to attend two additional focus groups. This was partly to increase the critical interaction in the data gathering and analysis phase, and also to contribute to ensuring data of greater depth. The data analysis was guided by a qualitative interpretive approach [37], which was strengthened by critical hermeneutic principles [34] and a humanizing framework [4].

### Participants

Two publicly financed NHs are included in this study. NH 1 specializes in dementia care, and NH 2 includes residents with various diagnoses, functional abilities, and care needs. Fifty residents live at NH 1, while NH 2 has 45 residents. Relatives of all residents in these NHs received written information and were invited to participate in this study without further selection criteria. The written information, and informed consent form, have previously been disseminated by our threefold research project [33]. The HMMs in the two NHs strengthened the recruitment process by directly encouraging relatives (in person and by telephone) to participate in the study. The number and size of the focus groups were adjusted to enable participation.

### Data gathering

Five focus groups were moderated by the first author (TAH) during April and May 2019. In addition to the first author, an assistant moderator participated in each focus group. The assistant moderator’s tasks were to conduct audio recordings and to write notes that described visual signals as well as group dynamics [38]. Each focus group meeting lasted for about 1.5 h and was conducted in a shielded meeting room at the NH. The number and distribution of participants is described in more detail in Table 1.

**Table 1:** Participants’ characteristics (*n* = 18)

Timeline Participants’ Characteristics	April 2019 Focus group 1	Focus group 2	Focus group 3	In total, First focus groups	May 2019 Focus group 4	Focus group 5	In total, Second focus groups
Number of participants	5	10	3	18	6	4	10
Women	3	6	2	11	5	3	8
Men	2	4	1	7	1	1	2
Spouse	1	3	2	6	1	3	4
Adult child	3	7	1	11	4	1	5
In-law	1	0	0	1	1	0	1
NH 1	2	9	2	13	2	3	5
NH 2	3	1	1	5	4	1	5
Age	47–73 years	34–80 years	53–90 years	34–90 years	47–68 years	56–80 years	47–80 years
Experience as relative of NHs	2–4 years	1–7 years	1–6 years	1–7 years	2–6 years	1–4 years	1–6 years

The semi-structured interview guide [33] that had been prepared for the first three focus groups contained open-ended questions, representing four topics: 1) participant characteristics; 2) NH quality; 3) leadership development; and 4) changes in NH quality after leadership development. The questions were framed in order to stimulate dialogue and reasoning from a critical and reflective perspective. The two additional focus group meetings utilized an interview guide that was designed to elaborate and explain data that had already been gathered from the same participants and for the same topics. Both interview guides have previously been disseminated [33]. The number of five focus groups were based on adaption to data saturation, as it was considered that the themes that emerged were repeated instead of new knowledge being added [39]. Of the same reason, there were not conducted any follow-up focus groups for this secondary analysis. The recorded data and notes from all focus groups were transcribed systematically and consistently, resulting in a total of 116 pages of verbatim text. Personal information was anonymized [40].

### Data coding and analysis

The transcribed text from the previous study became the focal point for re-interpretation based on the new research question. Providing an initial sense of the whole, the transcribed text was read repeatedly by all three authors. The transcripts from each focus group meeting were then condensed into units of meaning by the first author (TAH). This shortening process was designed to preserve the core meaning. Guided by a humanizing framework [4], the condensed units of meaning were further abstracted and sorted into themes and subthemes [37], specifically searching for knowledge that met the study’s aim. The analytical process was driven by critical reflection in a back-and-forth movement, according to the hermeneutical circle [35], that included all three authors. This movement went from transcribed text from each individual focus group to the transcribed text from all focus groups as a whole, and from the three authors’ pre-understanding to a shared new understanding [35]. The interpretation ended when the three authors agreed that good gestalt was reached without logical contradictions [37].

## Results

The participants (described by characteristics in Table 1) were 18 volunteer relatives, representing 16 of a total of 95 residents in the two NHs. Eleven of the 18 participants were women (68%). The participating relatives are mainly adult children (68%), but also spouses and in-laws. The youngest participant was 34 years old, and the oldest was 90 years old. All participants had more than one year of experience as being a relative of a resident in a NH.

The participating relatives’ experiences of influencing the dignified care of residents of NHs are illustrated as one main theme: preventing missed care when dignity is at stake. This main theme has two subthemes, which are elaborated with regard to the six existentials in this study’s humanizing framework [4].

### Preventing missed care when dignity is at stake

The main theme, preventing missed care when dignity is at stake, illustrates how the participating relatives repeatedly chose to take actions that they experienced temporarily safeguarded the residents’ dignity and identity. These choices were made when the relatives considered situations in the NHs as either being undignified or representing a risk of indignity. The actions of the relatives entailed either pointing out the situation to health personnel or the HMM, or actively compensating themselves for what they considered to constitute a deficiency. This pattern also became clear during the focus groups, when the participating relatives repeatedly gave each other feedback on topics that they thought the other participants should not abandon, or when they encouraged each other to raise issues and concerns with health personnel or HMMs. During one of the focus groups meetings, a relative expressed a clarifying moment, and noted to himself that, after the interview, he would complain about the lack of follow-up for his wife by a physiotherapist.

In contrast to how the relatives described the ensuring of dignity as the purpose of pinpointing or compensating, the three initial focus groups also critically discussed how these same actions also seemed to lead to indignity. The indignity here was explained as occurring when the present relatives’ interventions created an inequality with regard to the dignity of other residents, who did not have present relatives. When residents with present relatives seemed to be given priority, the needs of other residents could possibly be at risk of receiving lower priority. The main theme of this study has two, more detailed, subthemes 1) pinpointing to prevent missed care; and 2) compensating when dignity is threatened.

### Pinpointing to prevent missed care

The first subtheme, pinpointing to prevent missed care, refers to the relatives’ experiences of needing to be continuously present in order to actively prevent situations where deviations and quality failures threatened the dignity of their loved ones. The relatives described such situations that they had either observed themselves or had been told about by health personnel employed in the NHs. The relatives illustrated how they had questioned dignified care, for example, when they experienced that the resident had not been helped out of bed, had been left alone, or had been prohibited from participating in the activities offered. These findings confront the humanized existentials of inter-subjectivity, mood, identity, and embodiment.

Furthermore, the participants described how they had requested that their loved ones should receive their preferred food, avoid unhealthy food, and have their individual needs supported with regard to dental health care. Relatives also suggested QIs, reported deviations, and requested medical supervision, screening, and medication assessment. One example of this was provided when it was experienced that a resident had been isolated in his bed in the NH, without oversight by health personnel, for extended periods of time. This (cf. spatiality) was pinpointed by the resident’s daughter. Following her feedback to the HMM, a list appeared in the room, which the health personnel signed to confirm that they had been there every 15 min. The relatives checked the list when they visited, and the daughter depicted that she was satisfied that the quality of the service had improved. In contrast to this example, several participants described the inter-personal relationship in the NHs as being characterized by a feeling of caution with regard to how they expressed themselves, as feedback given to health personnel was often received negatively. The relatives described how they were afraid that their feedback could harm the inter-subjectivity between their loved ones and the health personnel, who might develop a negative attitude towards them. In the fifth focus group meeting, participant 5 (the son in-law of a resident) explained how he experienced the health personnel’s resistance to feedback:

…but they do not like it, if you question something… There are many who do not like it when I ask: Why does it smell like pee inside the room now? Then there is a diaper in the trash bin that is wet with pee. It should not be lying there. It must be carried away. And some do and some do not. And they get just as angry every time I ask them. So the middle manager, she has called me twice. And one time she wondered if I was annoyed; No, I said, I am not annoyed, I am overwhelmed and frustrated… it smells so much of urine in that room and in that bed… it must not be like that again, because it does not work in my head… it is such an indignity...

These empirical results form a contrast in this subtheme when the participating relatives describe their experiences of pinpointing missed care as constituting a control function for ensuring the provision of dignified care; however, this contributes to indignity when it comes to what could be considered isolation for those residents without the support of present relatives. The focus groups had several critical discussions about how those residents who did not have relatives to perform this control function received services of lower quality, and that this difference in inter-subjectivity, mood, and identity was even recognized by the health personnel in the NHs. In the first focus group meeting, participant 2 (the daughter of a resident) stated that:

... it is not because my dad is different to everyone else, because I expect everyone to get the same treatment. So, then, when they [the health personnel] are able to say… ..Yes, we observe who [relatives] runs in the hallways here, so we take that into account… then I think: Is there really such a difference between people? Everyone should have equal follow-up. That’s my opinion. Whether they have present relatives or not…

When summarizing this first subtheme, the relatives’ descriptions provides examples that imply that their presence is experienced as influencing five existentials of dignified care in NHs: spatiality, inter-subjectivity, mood, identity, and embodiment. Their influence on temporality is not illustrated in this subtheme.

### Compensating when dignity is threatened

In the second subtheme, the participating relatives elaborated on how they advocated the individual needs of the resident but compensated themselves when they experienced that dignified care was threatened. Their interactions concerned the existentials of the residents’ identity, mood, embodiment, temporality, and spatiality, when they offered examples of how they made and brought special food, facilitated alternative meals, helped the residents with meals, and brushed the resident’s teeth. The relatives brought and took care of clothes, and followed up appointments with the specialist healthcare, such as accompanying the resident on visits to medical specialists, and ensuring that any necessary follow-ups were undertaken.

A consistent challenge to dignified care, which was highlighted by the participants in all five focus groups, was experiences of inactivity and isolation. Here, relatives described how they tried to compensate in different ways. Relatives’ associations and other voluntary efforts contributed to the prevention of isolation, and added to mood, identity, and inter-subjectivity by, for example, the preparation and serving of shared traditional meals or by arranging music evenings. Relatives also broke the spatiality by taking the residents out of the NH, transporting them from their own rooms to group activities, or adding to the inter-subjectivity by socializing with other residents. Relatives experienced that they ensured the residents’ safety when health personnel were busy with other individual residents. In the second focus group meeting, participant 10 (the husband of a resident) explained:

…because I sit there often… I am there almost every day… often I am there more than once a day. So I have spent many hours in the nursing home... I often sit and watch when the nurses go into a room and take care of someone, because the risk of falling for many of those who are left is so high that they should not be alone at all.

The relatives also explained how they initiated everyday activity in the NHs, by, for example, playing music, singing, dancing, joking, or playing with a ball, thereby adding to inter-subjectivity, mood, and identity. In contrast to their descriptions of good intentions, the relatives generally experienced that such initiatives were not welcomed by health personnel, but were rather received as negative and disturbing. They therefore described these initiatives as being undertaken in spite of, and/or hidden from, the health personnel. In the first focus group meeting, participant 1 (the daughter of a resident) provided an example of this:

... Well, this week, there were such young girls at work and, they said nothing, and I had grandchildren with me, and they picked up a ball that was lying around, and began to roll it, and then one of the other residents, like, she got the ball and just kicked it, and dad, he raised his arms and wanted to join in… so… But they [the young health personnel] did not yell at us!

Summarizing this second subtheme, the participating relatives described experiences that included all six existentials in dignified care – spatiality, temporality, inter-subjectivity, mood, identity, and embodiment – and how they aimed to prevent suffering in the same areas.

## Discussion

This study aims to critically discuss relatives’ experiences of influencing the dignified care of residents of NHs. Our previously stated understanding of dignity and dignified care is based on Galvin and Todres’ [4] existential description, which is linked to the affirmation that every human being has inherent value. This is an understanding that also corresponds to health personnel’s ethical guidelines [6]. However, the results of this study expand this understanding by critically discussing how relatives understand dignity in NHs, and more specifically, how the experiences of their presence influence the dignified care provided for their loved ones. The results from this study support previous research when descriptions are provided of how relatives experience indignity in NHs [2, 11] and a need to be present in order to ensure the resident’s dignity [12]. This study adds to this knowledge by critically discussing the experiences of what the presence of relatives entails in NHs.

Galvin and Todres’ [4] descriptions of six existentials – spatiality, temporality, inter-subjectivity, mood, identity, and embodiment – offer a humanizing framework in which to understand dignified care. The results that form this study’s main theme, preventing missed care when dignity is at stake, bring together all six existentials when it is shown how relatives choose to take an active role, with the aim of ensuring that their loved ones receive dignified care. None of the six existentials dominates in these results; all are present in different ways. However, in the first subtheme, pinpointing to prevent missed care, only five of the six existentials are represented.

Temporality is added when it comes to the second subtheme, compensating when dignity is threatened. Høy et al. [9] have described elements of temporality as parts of maintaining dignity in NHs when the residents are involved as integrated members of society. However, this study provides new knowledge by demonstrating how relatives include all six existentials when they themselves compensate. This could be understood as their awareness of temporality as an existential part of the residents’ dignified care [4]. In addition, the results entail that relatives exclude temporality when it comes to pinpointing indignity to health personnel or HMMs. This knowledge provides more substance to the descriptions of NHs as institutionalized contexts that have a negative influence on the quality of life of older persons. There are few descriptions where older persons experience an opportunity to change this reality [20].

The results from this study explicate how the participating relatives experience repeatedly pointing out or compensating for deviations and quality failures when they feel that the dignity of their loved ones is threatened. These results are interesting when seen in the context of our primary analysis of the empirical data that is re-used in this study. The knowledge from the previous study explains how HMMs in NHs continuously supervise and compensate with the same aim as the relatives in this study: to develop or compensate [32] in order to prevent missed care. This connection may indicate that it is reasonable to question whether it is the case that relatives are trying to compensate for a lack of management. It may also be interesting to investigate further whether this indicates a quality failure in NHs that we lack the structural measures to address.

Lindwall and Lohne [11] describe indignity as being related to ignoring the needs of residents, and they point out that indignity in the care of older persons involves unethical attitudes on the part of the caregiver. This study, however, nuances this understanding, as unethical attitudes among health personnel are rarely emphasized; on the contrary, the results illustrate how relatives largely explain indignity in terms of factors that are beyond the health personnel’s control.

The NHs included in this study are part of what are described as the most service-intensive and universal benefits that are established by the welfare state model [17]. At the same time, medical developments, political priorities, and an increasing number of older persons have put this model under pressure [21]. In the critical discussions in the focus groups, there were several examples of where the participants claimed that indignity must be accepted. This view was based on an understanding of the NH’s difficult situation, with a lack of resources, and residents with complex needs. Relatives even described that their efforts to secure dignified care for their loved ones also contributed to indignity by resulting in differences between ways in which the residents are treated. This can be understood as what Habermas [34] explains as the system’s colonization of the lifeworld, where system imperatives based on, for example, power and money are what govern at the expense of individual knowledge and norms.

The critical reflection in the focus groups may be further described as an example of an inter-subjective process that ends in what can be seen as a form of communicative rationality [34]. Although this was toned down, the discussions regularly moved towards the viewpoint that indignity could not be accepted in any form, and that relatives also observed a lack of competence and unethical attitudes among the personnel. Despite equally distributed resources, a large degree of variation was described between employees, between units, and between the two NHs. These results support the research of Sworn and Booth [8], where the closest factors connected to missed care are acuity, complexity, amount of care, and organizational factors, although the connection to inadequate staffing is less well-evidenced.

Facilitating active relatives who are involved, participate and co-determine is what legislations, regulations, and other national guidelines require of health personnel [14, 26, 27]. It is described how relatives incorporate four different roles when they visit a NH [24]. However, this study expands our knowledge about these roles related to dignity, indignity, and dignified care, not least by adding knowledge about the challenges that relatives experience in the fulfillment of these roles. This study shows how the relatives’ critical observations are experienced as unpopular when communicated to health personnel or HMMs. Feedback from relatives is also generally experienced as not leading to a change in practice. At best, it results in temporary changes and only relates to the resident who has the support of present relatives who point out an indignity. The same negative response from health personnel is described when relatives actively offer to contribute practical help for the residents. As a contrast to how health authorities repeatedly highlight the need for help from volunteers [22], this study reveals that relatives experience their offers of feedback and voluntary support as a waste of time.

## Strengths and limitations

One of the strengths of this study is that it is (to our knowledge) one of the first studies that critically discusses relatives’ experiences of influencing dignified care provided in NHs in relation to the six existentials in a humanizing framework [4]. The fact that this study was designed and conducted in collaboration between three Scandinavian authors with different professions and academic backgrounds, as well as a top manager in a rural northern Norwegian municipality, strengthens the critical discussion. Furthermore, participation in this study provided relatives with the opportunity to get together and share their experiences of the NHs. In the focus groups, the participants explained that no one had previously asked them to do this, and that it was positive for them to be able to share their experiences together. Participation in this study could also be assumed as empowering for the relatives, which was exemplified in the focus group discussions by statements that indicated that the participants felt strengthened by the support of other participants.

As a result of adjustments to enable participation, the number of participants in each focus group varied from 3–10, although the time spent in each individual group was the same. This difference provided the individual participants in the smallest groups with the opportunity for longer reflections than those in the largest. This did not seem to affect the discussions or the results, as there was no equal distribution of speaking time, and there were no major differences in the content of the transcribed material between the focus groups.

One limitation of this study, however, may be that it involves data from only five focus groups, with 18 participating relatives in a Norwegian NH context, which also differs from other contexts in other countries, as they are not related to the Scandinavian or Nordic welfare state model [17]. This knowledge therefore makes a limited contribution to an area that must be described as having a high level of complexity [23]. The findings cannot therefore immediately be generalized to other contexts. However, based on Kvale and Brinkmann [37], analytical generalization is a possibility; the results can be considered as ‘indicative’ or transferable, in relation to other similar situations or settings.

### Implications for policy, practice, and theory

The results from this study imply that we have untapped potential with regard to how relatives are integrated as part of a NH context that safeguards residents’ dignity. For healthcare personnel, it provides knowledge about how relatives experience their role in NHs, how they experience their interactions with health personnel, and which legislations, guidelines, and opportunities would be embedded in an improved collaboration with relatives. For managers and decision-makers, the knowledge from this study can contribute to the facilitation of targeted QIs in order to reduce the discrepancies between government guidelines and practical everyday life in NHs with regard to the involvement of relatives.

## Conclusions

The main conclusions of this study are that relatives experience a challenging situation when their loved ones move to a NH, becoming dependent on how health personnel safeguard their dignity when the relatives themselves cannot be present. In this context, relatives describe how they observe situations in the NH characterized by both dignity and indignity related to six humanizing existentials, and how they have different ways of dealing with situations of indignity. One way of dealing with these situations is to pinpoint the indignity to health personnel or HMMs; the other is to take measures into their own hands in order to maintain the dignity of their loved ones. However, the results in this study show that the participating relatives, despite their stated good intentions to safeguard dignity, experience being alienated in their attempts to transform what they describe as undignified and unacceptable practice into dignified care.

The user knowledge obtained by relatives with regard to their observations of dignity or indignity is, in contrast to national and international regulations, not experienced as being mapped and/or used in any form of systematic improvement work in the NHs in this study. This knowledge indicates that the implementation of knowledge-based practice in NHs, including the active application of user and relative knowledge, has the potential to contribute towards QIs with regard to dignified care.

## Data Availability

The datasets that were generated and secondary analyzed during the current study are not publicly available, due to the confidentiality afforded study participants, but they are available from the corresponding author on reasonable request.

## Abbreviations

HMM: Healthcare middle manager
NH: Nursing home
QI: Quality improvement
